# The accuracy of anatomic landmarks on the occlusal plane: a comparative study between conventional and 3D image method

**DOI:** 10.1186/s12903-024-05132-6

**Published:** 2024-12-02

**Authors:** Supak Kingrungpetch, Weerapan Aunmeungtong, Pathawee Khongkhunthian

**Affiliations:** Center of Excellence for Dental Implantology, Faculty of Dentistry, Chiang Mai University, Chiang Mai, 50200 Thailand

**Keywords:** 3D Image, Ala-tragus line, Occlusal plane, Virtual picture

## Abstract

**Background:**

To establish the occlusal plane, the conventional methods for facial analysis to gain accurate alignment of the occlusal plane are inadequate, while 3D technologies are an ideal diagnostic tool. The aims of this research are to compare the difference accuracy of anatomic landmarks on the occlusal plane and ala-tragus line between the conventional clinical method and 3D image method in both non-orthodontic and orthodontic treatment volunteers.

**Methods:**

A total of 44 volunteers (22 non-orthodontic and 22 orthodontic treatment volunteers) with normal occlusion were selected. All volunteers received 2 operative methods for occlusal plane determination. In conventional method, the occlusal plane was defined by the fox plane line. The ala-tragus line was defined by the radio-opaque markers. In the 3D image method, the volunteers were recorded intraoral images, 3D facial images and CBCT images. A 3D virtual picture was created using EXOCAD® software. The occlusal plane was generated by the incisal and occlusal surfaces of the teeth. Both methods, the angles and distances between the occlusal plane and ala-tragus line were measured and compared statistically on both sides of each volunteer.

**Results:**

Both volunteers’ group, the mean angles and distances between the occlusal plane-ala tragus line in the conventional method were reported to be significantly greater than the 3D method (*P* < 0.05). The percentage difference of angles in conventional method were reported to be significantly higher by 13.61–21.58% (*p* < 0.05) compared to the 3D method. The percentage difference of distances in the conventional method were reported to be significantly greater than the 3D method by 4.73–7.51% (*p* < 0.05).

**Conclusions:**

Within the limitation of the study, it can be concluded that both conventional and digital methods for establishing the occlusal plane are not parallel to the occlusal plane. The occlusal plane and ala-tragus line in the conventional method and the 3D method were significantly different in terms of angles and distances in both non-orthodontic and orthodontic treatment volunteers. However, the deviation angle of both methods is approximately 13–20 degrees, which is clinically acceptable for occlusal plane establishment. The accuracy of both methods is still within the using in clinical implementation.

## Introduction

Occlusal plane determination in edentulous patients has been proposed in several studies [1–3]. According to the glossary of prosthodontic terms 2017 (GPT-9), the occlusal plane or curve of occlusion is defined as the average plane generated by the incisal and occlusal surfaces of the teeth, wherein it is usually not a plane, but rather the planar mean of the curvature of these surfaces. The surfaces of wax occlusion rims molded to assist in the configuration of denture teeth, or a flat or curved mold used in placement of dentures [4]. The occlusal plane can be configured using features in both the mandibular and maxillary arches [5]. In the mandibular arch, several landmarks can be utilized to align the occlusal plane such as retromolar pad, the lateral border of the tongue or buccinator groove [6–8].

**Fig. 1:**
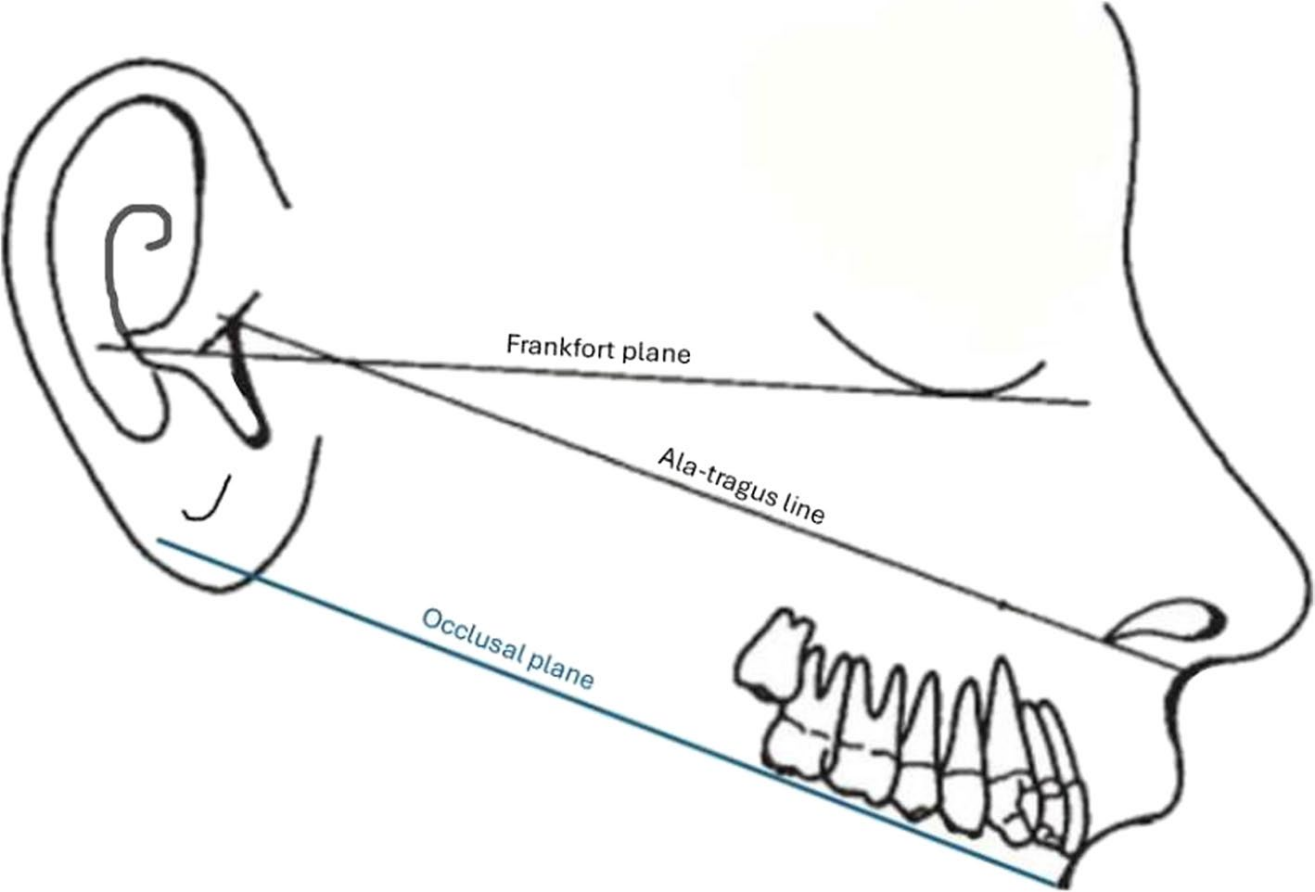
The Occlusal plane and the Camper plane (ala-tragus line)

In the maxillary arch, an ala-tragus line is the most popular marker for establishing the occlusal plane [9, 10]. An ala-tragus line is defined as a line extending from the lower border of the ala of the nose to the upper border on the tragus of the ear along with a third point on the opposite tragus [11]. When observed in the mid-sagittal plane, the ala-tragus plane should be aligned to the occlusal plane where the occlusal plane should be at an incline about 10 degrees in relation to the Frankfort horizontal plane [4] (Fig. 1).

The correct alignment of the occlusal plane is critical for a denture prosthesis [12]. The conventional methods of facial analysis for the proper alignment of the occlusal plane use either a two-dimensional (2D) photography or a fox plane device to evaluate the projection of distances and angles [13]. The fox plane device is used to check the parallelism in anteroposterior direction between the occlusal plane and ala-tragus line [14]. The cephalometric radiographs have been widely used in alignment of the occlusal plane and ala-tragus line, but these radiographs are based on the superimposition of the left and right sides [15]. While improvements and evolutions in optical scanning have led to a change from two-dimensional (2D) to the increasingly popular three-dimensional (3D) technology used in digital dentistry today [16], the management of dental treatment has been disrupted by the usage of 3D, for example, the radiological diagnosis and planning has been changed to cone beam CT-scan as well as the digital impression technique [17].

Three-dimensional (3D) innovations have proven to be the important diagnostic tools for facial examination as the graphics can be manipulated, turned, and magnified to provide a more realistic impression [15]. The feature points of a face can be electronically documented using face scanning while features of the oral region can be documented electronically using an intraoral scanner [18]. Moreover, dental cone beam computed tomography (CBCT) pictures provide volumetric data on jawbones and teeth, assisting in diagnostic and therapeutic management for total denture prosthodontics or oral physiotherapy [19]. Furthermore, data from CBCT, intraoral scans, and facial scans can be layered to create a "virtual picture" [20, 21]. Joda and colleagues reported systematic review of digital superimposition innovations to create virtual patients, superimposition of data on the facial soft tissue, bony structures and intraoral scan is currently a possible method to create a virtual patient under static conditions for improving results in diagnosis and treatment management [22].

Orthodontic treatment seems to have an effect on occlusion and masticatory function [23]. In clinical practice of orthodontic treatment plan, the goal of orthodontic treatment was to achieve normal occlusion. Bony landmarks in conventional method have been utilized in lateral cephalometric radiographs for occlusal plane orientation [24]. A systematic review of stable cranial parameters to assess the orientation of the occlusal plane. The interpupillary plane does not represent a reliable reference for evaluating the orientation of the occlusal plane. In dentate patients, The Frankfurt plane represents a more stable reference for occlusal plane determination [25]. Moreover, a systematic review of the effects of orthodontic treatment with four premolar extractions compared to non-extraction treatment. The evidence suggests that orthodontic treatment with four premolar extractions has no specific effect on the skeletal vertical dimension [26]. Currently, bony landmarks in 3D method provide accurate sagittal assessment. However, there is a limitation between soft tissue landmarks and the occlusal plane in both methods.

Presently, conventional methods for facial analysis to gain accurate alignment of the occlusal plane are inadequate, while 3D technologies are an ideal diagnostic tool [20]. As a result, the aims of this research are to compare the conventional clinical approach with the 3D imaging approach in terms of occlusal plane and ala-tragus line anatomic landmark accuracy in non-orthodontic treatment volunteers and to compare the difference accuracy of anatomic landmarks on the occlusal plane and ala-tragus line between the conventional clinical method and 3D image method in orthodontic treatment volunteers.

## Methods

This study was followed by Helsinki declaration and approved by the Human Experimentation Committee, Office of Research Ethics, Faculty of Dentistry, Chiang Mai University with certificate of human research number 16/2022.

### Sample size calculation and volunteer selection

According to G*power analysis (Düsseldorf University, Germany), the significance level (α) was set at 0.05 and power of test (1-β) was set at 80%. Sample size was calculated, minimum 16 volunteers in each group were required. All volunteers received study information and signed their informed consent for study participation. Volunteer selection was followed by inclusion and exclusion criteria as shown in Table 1.

**Table 1:** Inclusion and exclusion criteria

Inclusion criteria	Exclusion criteria
Volunteers between 20–40 years old	Syndromic craniofacial anomaly
Volunteers with permanent dentition (at least 24 teeth)	Traumatic facial deformity
Volunteers with a stable centric occlusion and molar classification I	Temporomandibular joint diseases
Volunteers in good health	Tooth mobility and tooth loss
Volunteers with a good attitude and patient’s compliance	Remaining deciduous teeth

### Study design

All volunteers received 2 operative methods for occlusal plane determination.

**Fig. 2:**
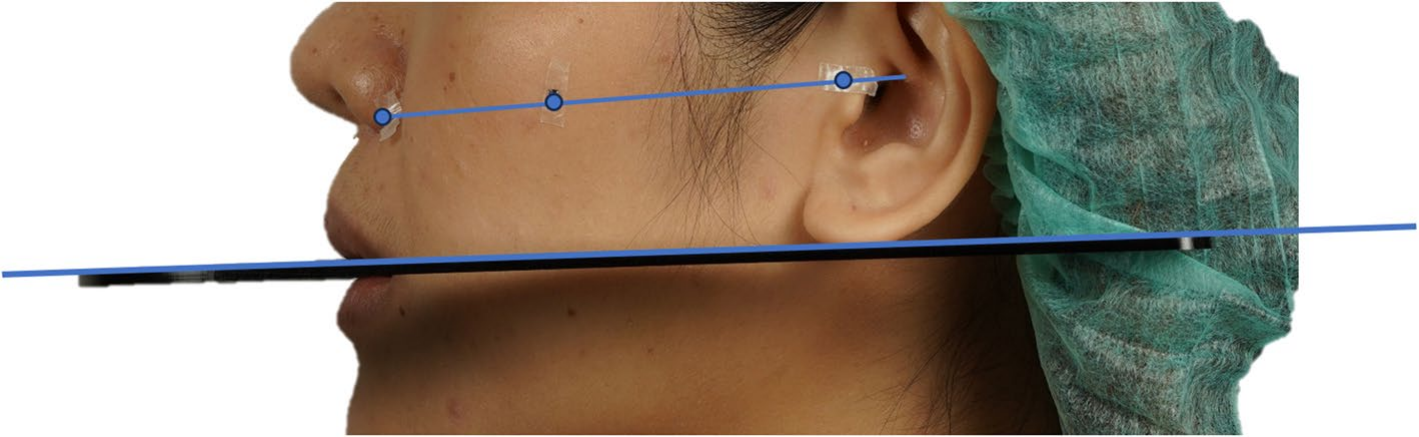
The line connecting lead foil radio-opaque marker refers to the ala-tragus line and the line of fox plane refers to the occlusal plane

**Fig. 3:**
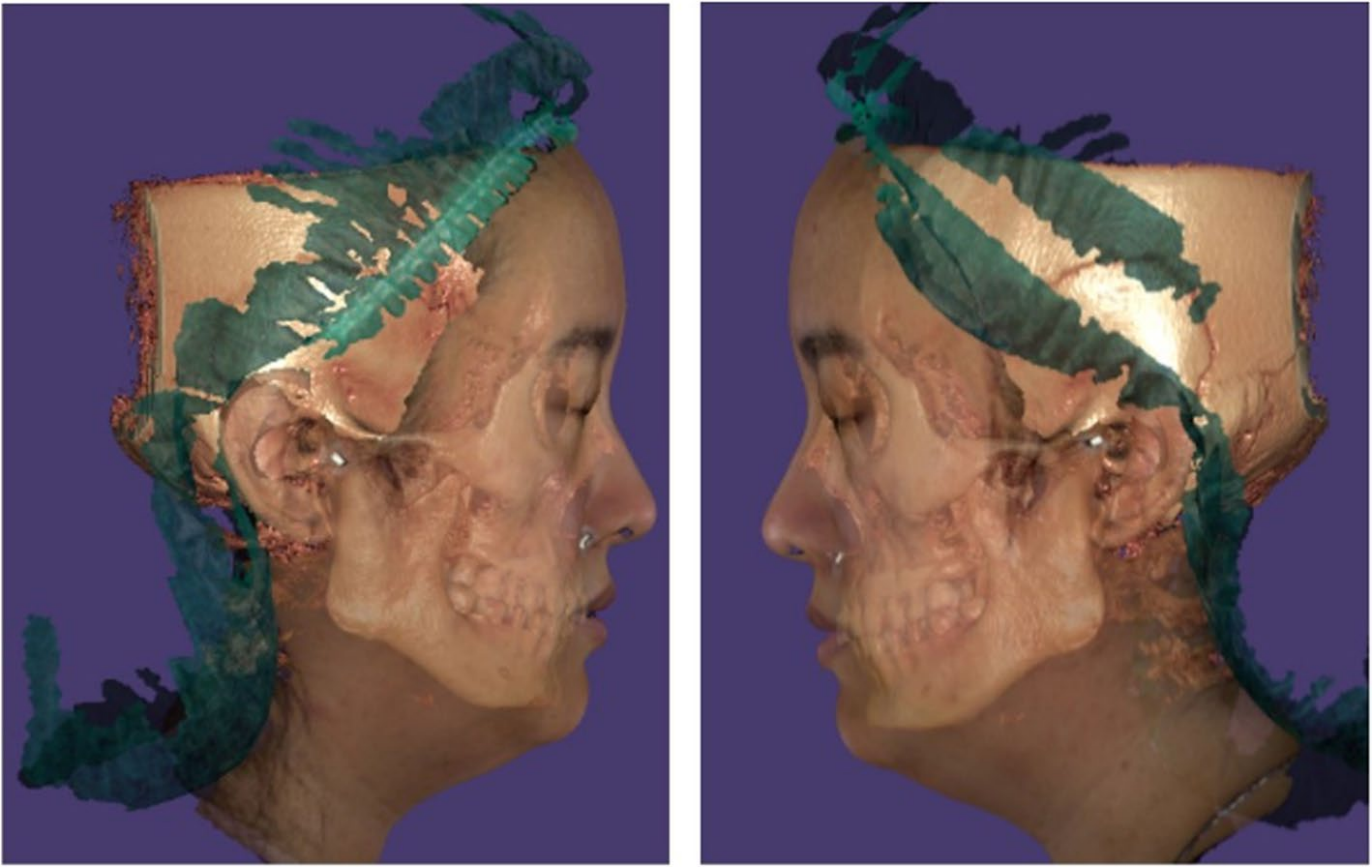
A best-fit alignment of 3D image with EXOCAD® software

1. Conventional method: The upper tragus, the middle point between upper tragus-lower border of the ala of the nose and the lower border of the ala of the nose were indicated with a lead foil radio-opaque marker on each of the volunteers. The line connecting the lead foil radio-opaque marker was referred to as an ala-tragus line. The volunteers were asked to hold a fox plane 3 times. The fox plane was positioned intraorally, touching the incisal edges of the upper incisors as well as the cusps of the left and right upper first molars. The occlusal plane was defined by the fox plane line (Fig. 2). Left and right profile photographs were taken with a digital camera using 3 images. In this study, a Sony A7III (Sony Corporation, Tokyo, Japan) with a resolution of 25.3 megapixels was used, which was suitable for computer analysis. The volunteers were remained naturally erect and upright, with straight backs and heads facing forward.

2. 3D image method: The Trios 3® intraoral scanner (3Shape A/S, Copenhagen, Denmark) was used to collect intraoral 3D images and bite registration 3D images. For measurements, scan data files were stored as stereolithography (STL) files. A facial scanner (EinScan Pro 2X Plus® (EP + ®) (Shining 3D, Hangzhou, China)) was used to collect 3D facial images during the same appointment. The lead foil radio-opaque marker was used to mark the volunteers on the upper tragus, the center between upper tragus-lower border of the ala of the nose and the lower border of the ala of the nose. Volunteers were scanned in a neutral-facing position with closed lips and bite registration. Scan data files were saved using the polygon file format (PLY) for measurements. CBCT images were obtained using a DentiiScan 2.0® (NECTEC NSTDA, Pathum Thani, Thailand) with a voxel resolution of 0.4 mm. The Digital Imaging and Communications in Medicine (DICOM) format was used to record all image data. All STL files, PLY files, and DICOM files were correlated using a best-fit alignment with EXOCAD® software 3 times (Exocad America Inc., Massachusetts, USA) (Fig. 3).

### Clinical outcome variables

**Fig. 4:**
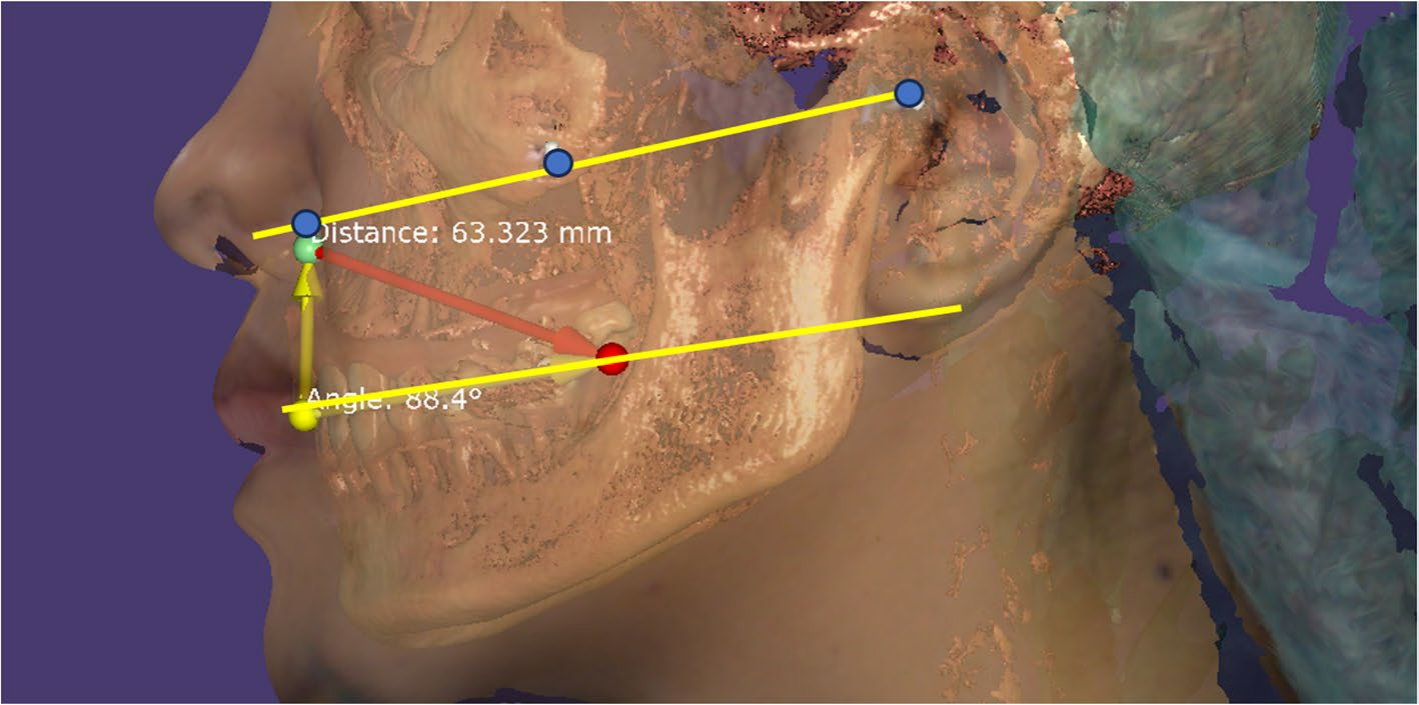
The angle of landmarks line

**Fig. 5:**
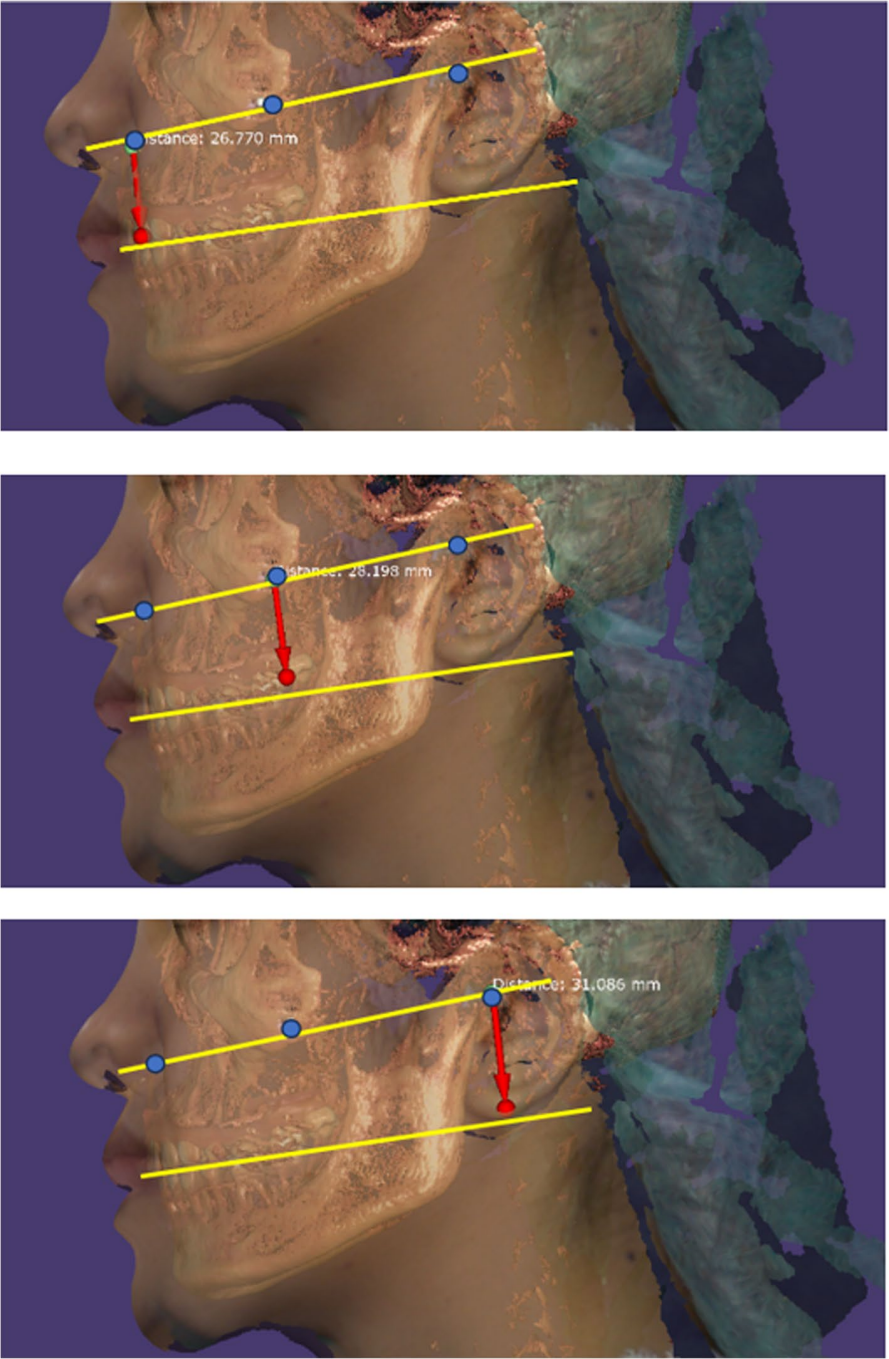
The distances of landmarks line

1. The difference between the angle of landmarks line: the comparison between the conventional method and 3D image method using the mean angle between the right ala-tragus line and right occlusal plane, as well as the left ala-tragus line and left occlusal plane. The computer programmed Screen Protractor was utilized to conduct measurement of the conventional method and EXOCAD® software was utilized to conduct measurement of the 3D image method (Fig. 4).

2. The difference between landmark line distances: the comparison between the conventional method and the 3D image method using the mean distances between the right ala-tragus line and right occlusal plane, as well as the left ala-tragus line and left occlusal plane. The measurements were taken on both sides at three locations, which included the lower border of the ala of the nose, the center, and the upper tragus. The digital ruler was utilized to conduct measurement of the conventional method and EXOCAD® software was utilized to conduct measurement of the 3D image method (Fig. 5).

### Statistical analysis

The statistical analyses were made with the SPSS Statistics 26 software (SPSS Inc., Chicago, Illinois). The normal distribution of data was tested by the Kolmogorov–Smirnov and Shapiro–Wilk normality test in each variance.

All data parameters of both methods were evaluated. The statistical analysis was performed using a dependent sample T-test for comparison. Differences at *p* < 0.05 were considered statistically significant.

All data parameters of both volunteers were evaluated. The statistical analysis was performed using an independent T-test for comparison. Differences at *p* < 0.05 were considered statistically significant.

The reliability of study was presented in inter-rater and intra-class correlation coefficient.

Patient demographic information was reported with descriptive statistics. Gender proportion was reported with Chi-square and Fisher’s Exact Test.

## Results

According to the angle and distance of both methods were normally distributed (Shapiro–Wilk test).

The reliability of study was presented in inter-rater and intra-class correlation coefficient.

The intra-rater reliability was 0.942 (0.542–0.989) and inter-rater reliability was 0.863 (-0.14–0.97).

The demographic information was presented in Table 2. A total of 44 volunteers (12 male and 32 female) aged between 20 to 40 years were participated in this study, including 22 volunteers with history of orthodontic treatment and 22 volunteers without history of orthodontic treatment. Gender proportion in non-orthodontic treatment volunteers and orthodontic treatment volunteers were not found to be significantly different (*p* = 0.31, Fisher’s Exact Test).

**Table 2:** Demographic information

Volunteers	n	Male	Female
Non-orthodontic treatment volunteers	22	8	14
Orthodontic treatment volunteers	22	4	18
All volunteers	44	12	32

**Fig. 6:**
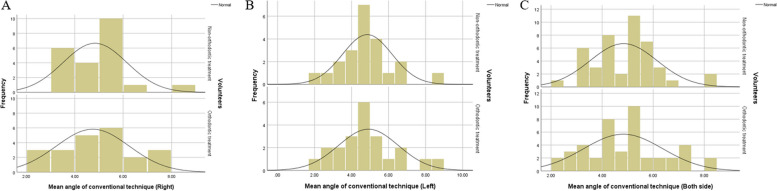
Histogram of angles between the occlusal plane-ala tragus line in conventional technique. **A** Right side (**B**) Left side (**C**) Both side

**Fig. 7:**
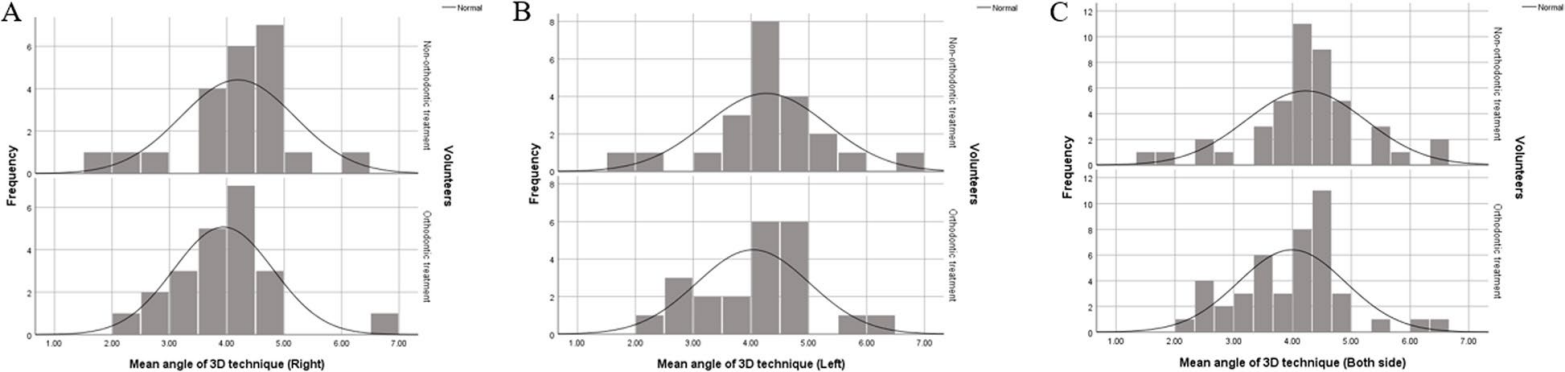
Histogram of angles between the occlusal plane-ala tragus line in 3D technique (**A**) Right side (**B**) Left side (**C**) Both side

The mean angles between the occlusal plane and ala tragus line in each method were presented in Table 3 and Figs. 6 and 7. The mean angles in the conventional method were reported to be significantly greater than that in the 3D method (*P* < 0.05). The percentage difference of angles in conventional method were reported to be significantly higher by 13.61–21.58% (*p* < 0.05) compared to the 3D method.

**Table 3:** Comparison of angles between the occlusal plane-ala tragus line

Subjects	Angle between ala tragus—occlusal plane	Mean angle of conventional technique ± SD (degree)	Mean angle of 3D Technique ± SD (degree)	Percentage difference**	*P*-values
All volunteers	Right	4.78 ± 1.40	4.06 ± 0.92	17.73	0.001*
	Left	4.87 ± 1.46	4.14 ± 1.01	17.63	0.001*
	Both side	4.83 ± 1.42	4.10 ± 0.96	*17.80*	0.000*
Non-orthodontic treatment volunteers	Right	4.83 ± 1.32	4.19 ± 0.99	15.27	0.023*
	Left	4.84 ± 1.33	4.26 ± 1.05	13.61	0.021*
	Both side	4.84 ± 1.31	4.22 ± 1.01	*14.69*	0.001*
Orthodontic treatment volunteers	Right	4.47 ± 1.50	3.95 ± 0.86	13.16	0.027*
	Left	4.90 ± 1.61	4.03 ± 0.97	21.58	0.024*
	Both side	4.82 ± 1.54	3.98 ± 0.91	*21.10*	0.001*

^*^The mean difference is significant at the 0.05 level

^**^ $$\frac{\text{Percentage difference} = (\text{mean angles of conventional technique} - \text{mean angle of 3D Technique})*100}{\text{mean angle of 3D Technique}}$$

**Fig. 8:**
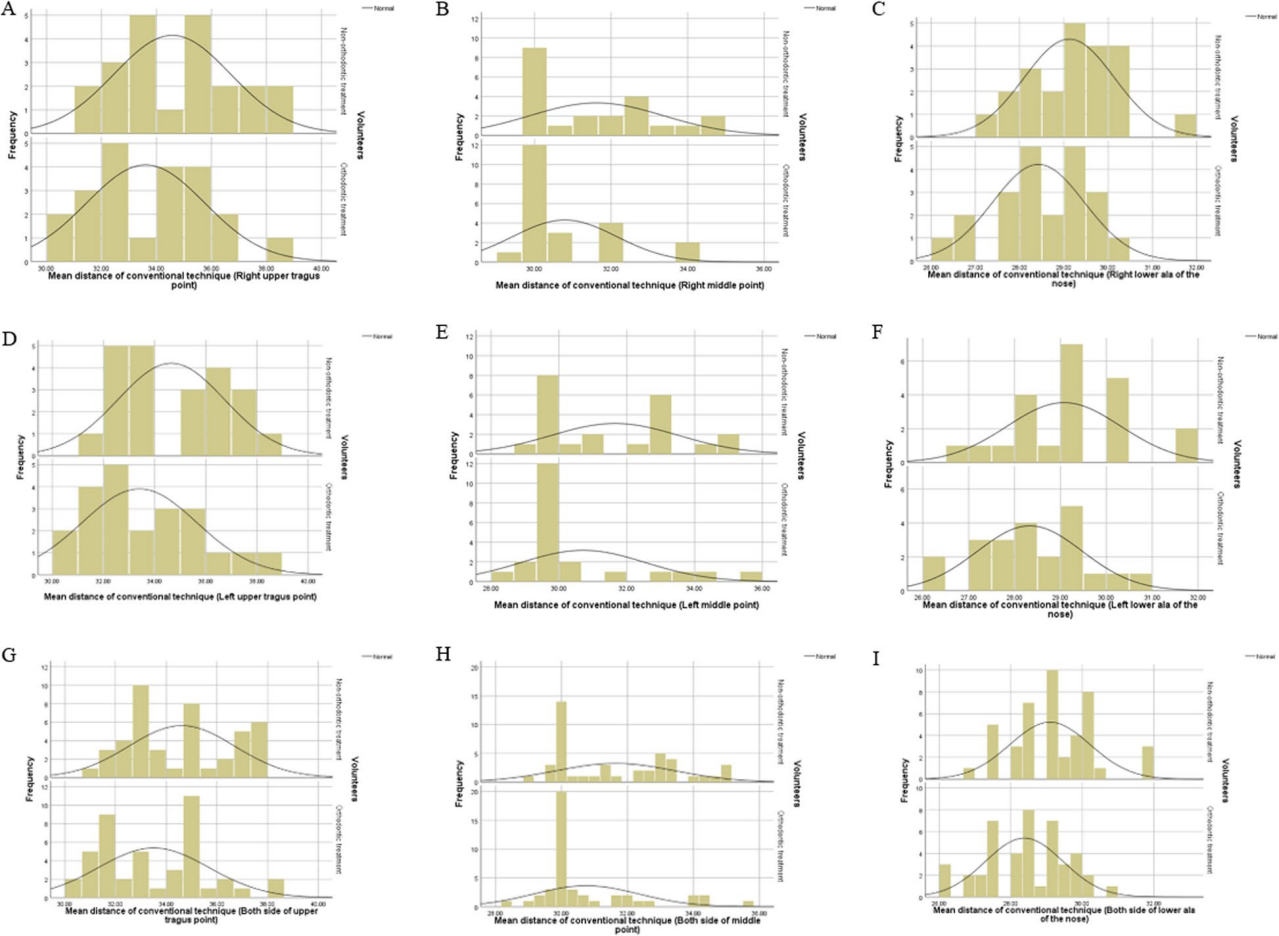
Histogram of distances between the occlusal plane-ala tragus line in conventional technique. **A** Right upper tragus (**B**) Right middle (**C**). Right lower ala of the nose (**D**) Left upper tragus (**E**) Left middle (**F**) Left lower ala of the nose. **G** Both side of upper tragus (**H**) Both side of middle (**I**) Both side of lower ala of the nose

**Fig. 9:**
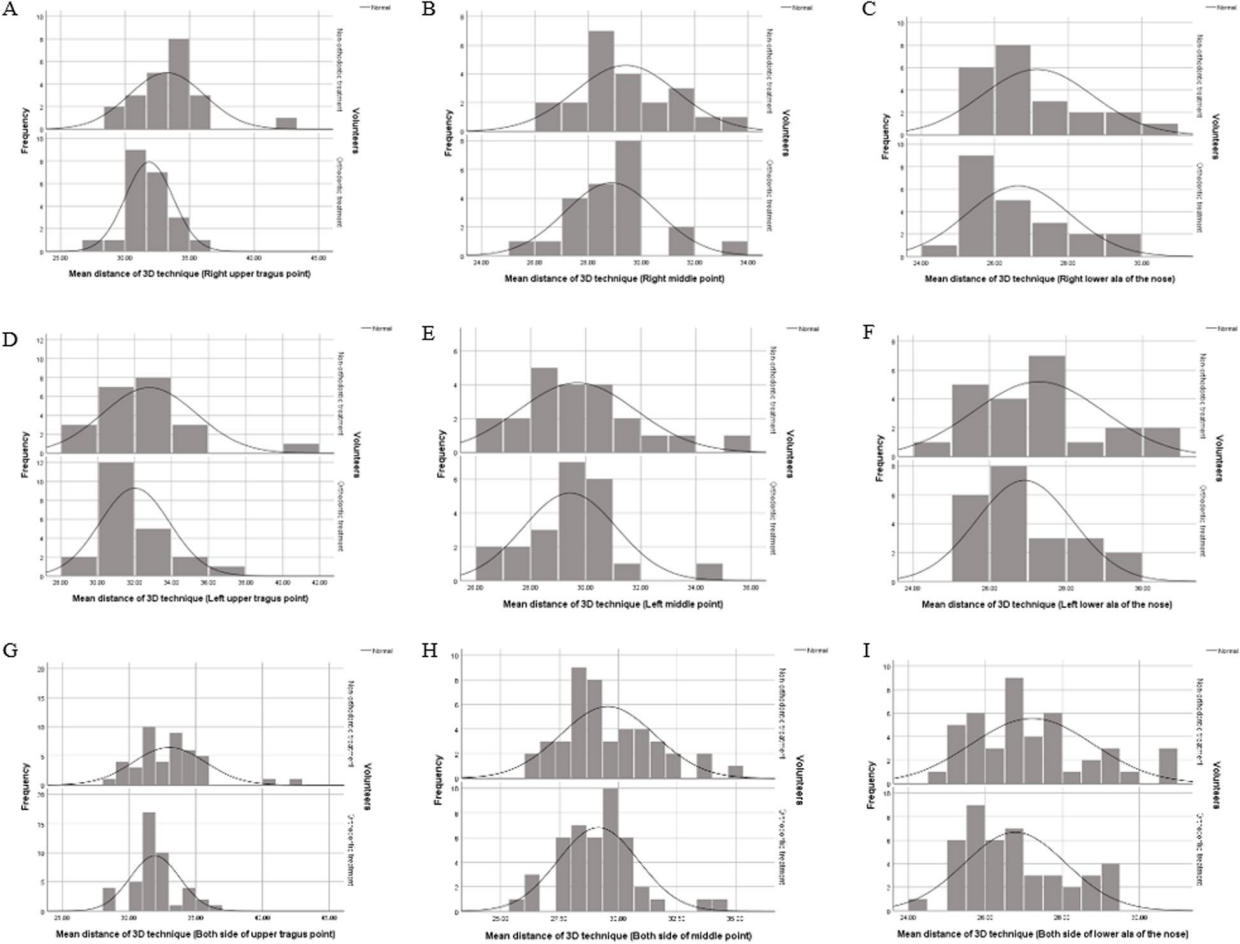
Histogram of distances between the occlusal plane-ala tragus line in 3D technique. **A** Right upper tragus (**B**) Right middle (**C**) Right lower ala of the nose. **D** Left upper tragus (**E**) Left middle (**F**) Left lower ala of the nose. **G** Both side of upper tragus (**H**) Both side of middle (**I**) Both side of lower ala of the nose

The mean distances between the occlusal plane and ala tragus line in each method were presented in Table 4, including the upper tragus, the center, and lower border of the ala of the nose. The mean distances in the conventional method were reported to be significantly greater than that in the 3D method (*P* < 0.05). The percentage difference of distances in the conventional method were reported to be significantly greater than the 3D method by 4.73–7.51% (*p* < 0.05) Figs. 8 and 9.

**Table 4:** Comparison of distances between the occlusal plane-ala tragus line

Subjects	Distance between ala tragus—occlusal plane		Mean distance of conventional technique ± SD (mm)	Mean distance of 3D Technique ± SD (mm)	Percentage difference **	*P*-values
All volunteers	Right	upper tragus	34.09 ± 2.16	32.55 ± 2.51	4.73	0.000*
		middle	31.20 ± 1.60	29.14 ± 1.81	7.06	0.000*
		lower ala of the nose	28.77 ± 1.07	26.89 ± 1.45	6.99	0.000*
	Left	upper tragus	34.03 ± 2.23	32.40 ± 2.24	5.03	0.000*
		middle	31.18 ± 1.90	29.55 ± 1.91	5.51	0.000*
		lower ala of the nose	28.70 ± 1.23	27.09 ± 1.48	5.94	0.000*
	Both side	upper tragus	34.06 ± 2.18	32.47 ± 2.36	*4.89*	0.000*
		middle	31.19 ± 1.75	29.35 ± 1.86	*6.26*	0.000*
		lower ala of the nose	28.73 ± 1.15	26.99 ± 1.46	*6.44*	0.000*
Non-orthodontic treatment volunteers	Right	upper tragus	34.57 ± 2.11	33.23 ± 2.92	4.03	0.029*
		middle	31.62 ± 1.75	29.41 ± 1.90	7.51	0.000*
		lower ala of the nose	29.12 ± 1.02	27.15 ± 1.50	7.25	0.000*
	Left	upper tragus	34.65 ± 2.08	32.79 ± 2.52	5.67	0.001*
		middle	31.65 ± 1.88	29.69 ± 2.13	6.60	0.000*
		lower ala of the nose	29.07 ± 1.23	27.28 ± 1.68	6.56	0.000*
	Both side	upper tragus	34.61 ± 2.07	33.01 ± 2.70	*4.84*	0.000*
		middle	31.63 ± 1.79	29.55 ± 2.00	*7.03*	0.000*
		lower ala of the nose	29.09 ± 1.12	27.21 ± 1.58	*6.90*	0.000*
Orthodontic treatment volunteers	Right	upper tragus	33.60 ± 2.14	31.86 ± 1.85	5.46	0.002*
		middle	30.78 ± 1.35	28.87 ± 1.72	6.61	0.000*
		lower ala of the nose	28.42 ± 1.03	26.64 ± 1.39	6.68	0.000*
	Left	upper tragus	33.40 ± 2.24	32.00 ± 1.89	4.37	0.010*
		middle	30.71 ± 1.84	29.42 ± 1.69	4.38	0.005*
		lower ala of the nose	28.33 ± 1.14	26.90 ± 1.25	5.31	0.000*
	Both side	upper tragus	33.50 ± 2.17	31.93 ± 1.84	*4.91*	0.000*
		middle	30.75 ± 1.60	29.14 ± 1.71	*5.52*	0.000*
		lower ala of the nose	28.37 ± 1.08	26.77 ± 1.31	*5.97*	0.000*

^*^The mean difference is significant at the 0.05 level

^**^ $$\frac{\text{Percentage difference} = (\text{mean distance of conventional technique} - \text{mean distance of 3D Technique})*100}{\text{mean distance of 3D Technique}}$$

The mean angles in the conventional method between non-orthodontic treatment volunteers and orthodontic treatment volunteers were presented in Table 5. The mean angles in both volunteers were not found to be significantly different (*p* > 0.05).

**Table 5:** Comparison of angles between non-orthodontic and orthodontic treatment volunteers in conventional technique

Angle between ala tragus—occlusal plane	Non-orthodontic treatment volunteers ± SD (degree)	Orthodontic treatment volunteers ± SD (degree)	*P*-values
Right	4.83 ± 1.32	4.47 ± 1.50	0.832
Left	4.84 ± 1.33	4.90 ± 1.61	0.893
Both side	4.84 ± 1.31	4.82 ± 1.54	0.96

The mean angles in the 3D method between non-orthodontic treatment volunteers and orthodontic treatment volunteers were presented in Table 6. The mean angles in both volunteers were not found to be significantly different (*p* > 0.05).

**Table 6:** Comparison of angles between non-orthodontic and orthodontic treatment volunteers in 3D Technique

Angle between ala tragus—occlusal plane	Non-orthodontic treatment volunteers ± SD (degree)	Orthodontic treatment volunteers ± SD (degree)	*P*-values
Right	4.19 ± 0.99	3.95 ± 0.86	0.379
Left	4.26 ± 1.05	4.03 ± 0.97	0.462
Both side	4.22 ± 1.01	3.98 ± 0.91	0.249

The mean distances in the conventional method between non-orthodontic treatment volunteers and orthodontic treatment volunteers were presented in Table 7. The mean distances in both volunteers were not found to be significantly different (*p* > 0.05).

**Table 7:** Comparison of distances between non-orthodontic and orthodontic treatment volunteers in conventional technique

Distance between ala tragus—occlusal plane		Non-orthodontic treatment volunteers ± SD (mm)	Orthodontic treatment volunteers ± SD (mm)	*P*-values
Right	upper tragus	34.57 ± 2.11	33.60 ± 2.14	0.139
	middle	31.62 ± 1.75	30.78 ± 1.35	0.085
	lower ala of the nose	29.12 ± 1.02	28.42 ± 1.03	0.055
Left	upper tragus	34.65 ± 2.08	33.40 ± 2.24	0.064
	middle	31.65 ± 1.88	30.71 ± 1.84	0.102
	lower ala of the nose	29.07 ± 1.23	28.33 ± 1.14	0.075
Both side	upper tragus	34.61 ± 2.07	33.50 ± 2.17	0.063
	middle	31.63 ± 1.79	30.75 ± 1.60	0.074
	lower ala of the nose	29.09 ± 1.12	28.37 ± 1.08	0.09

The mean distances in the 3D method between non-orthodontic treatment volunteers and orthodontic treatment volunteers were presented in Table 8. The mean distances in both volunteers were not found to be significantly different (*p* > 0.05).

**Table 8:** Comparison of distances between non-orthodontic and orthodontic treatment volunteers in 3D Technique

Distance between ala tragus—occlusal plane		Non-orthodontic treatment volunteers ± SD (mm)	Orthodontic treatment volunteers ± SD (mm)	*P*-values
Right	upper tragus	33.23 ± 2.92	31.86 ± 1.85	0.71
	middle	29.41 ± 1.90	28.87 ± 1.72	0.329
	lower ala of the nose	27.15 ± 1.50	26.64 ± 1.39	0.249
Left	upper tragus	32.79 ± 2.52	32.00 ± 1.89	0.247
	middle	29.69 ± 2.13	29.42 ± 1.69	0.653
	lower ala of the nose	27.28 ± 1.68	26.90 ± 1.25	0.413
Both side	upper tragus	33.01 ± 2.70	31.93 ± 1.84	0.32
	middle	29.55 ± 2.00	29.14 ± 1.71	0.315
	lower ala of the nose	27.21 ± 1.58	26.77 ± 1.31	0.159

## Discussion

The occlusal plane is important in prosthetic treatment planning [27] A proper occlusal plane alignment is a crucial parameter during the fabrication process of prosthesis [12]. Although the natural occlusal plane is curved due to the sagittal inclination of the teeth, a flat occlusal plane is practically formed as a reference for prosthesis construction [28]. Clinically, a common method to establish the tentative occlusal plane is by aligning it parallel to the ala-tragus line [29]. Kumar and colleagues [30] suggested that the Frankfort horizontal plane, ala-tragus line, and palatal plane can be used as reliable guides to establish the occlusal plane. An ala-tragus line is the most popular and extends from the ala of the nose to the tragus of the ear. The ala-tragus line is considered parallel to the occlusal plane, supported by evidence-based cephalometric radiographic analytical studies. According to the glossary of prosthodontic terms 2017 (GPT-9), in this study has shown that the ala-tragus line was defined as a line extending from the lower border of the ala of the nose to the upper border on the tragus of the ear along with a third point on the opposite tragus. The occlusal plane was defined as the average plane generated by the incisal and occlusal surfaces of the teeth [4]. The results of this study have shown that the mean angle in the conventional method was reported significantly greater than in the 3D method (*P* < 0.05), and the mean distance in the conventional method was reported significantly greater than in the 3D method (*P* < 0.05). Additionally, the percentage difference of angles in conventional method were reported to be significantly higher by 13.61–21.58% (*p* < 0.05) compared to the 3D method. The percentage difference of distances in the conventional method were reported to be significantly greater than the 3D method by 4.73–7.51%. (*p* < 0.05) in both non-orthodontic and orthodontic treatment volunteers.

Regarding the angle between the ala-tragus line and the occlusal plane, Khan and colleagues [11] conducted an evaluation on the parallelism of the ala-tragus plane in occlusal plane orientation. In their study, the angulation of the occlusal plane to Camper’s line was found to be4.25 degrees on right side and 4.50 degrees on left side, while Shrestha and colleagues [31] reported it as 5.18 degrees. Khan and colleagues [11] used the inferior border of the tragus as the posterior border of the ala-tragus line, while Shrestha and colleagues [31] used the center of the tragus as the posterior border of Camper’s line. Additionally, a study conducted by Raza and colleagues [32] reported the angulations of the occlusal plane to Camper’s line in dentate patients as 3.26 degrees and 2.34 degrees on right side and left side, respectively. Moreover, Nayar and colleagues [33] studied the angle between the ala-tragus line and the occlusal plane in dentate patients. There was no parallelism between the occlusal plane and ala-tragus positions. In our study, it was corresponding to this study that the ala-tragus line was not parallel to the occlusal plane on both sides due to the variations in the ala-tragus plane on both sides.

A number of studies [31–33] have used two-dimensional (2D) photography or a fox plane device to evaluate the 2D projection of distances and angles between the ala-tragus lines and the occlusal plane [13]. Presently, three-dimensional (3D) technology has proven to be an important diagnostic tool for facial examination [15]. Rosati and colleagues [34] conducted three-dimensional full-face reconstruction which the results showed that the ala-tragus line was not parallel to the occlusal plane. Additionally, with the result from Valério and colleagues [35] which reported a tomographic study of 27 well-preserved adult skulls. These results showed that the ala-tragus line was not parallel to the occlusal plane. Our study agreed with the result of Rosati and colleagues’ s study and Valério and colleagues 's study.

Regarding the distance between the ala-tragus line and the occlusal plane, Woelfel and colleagues [36] conducted a study and reported measurements in three locations: near the tragus, near the ala, and at the midpoint. They found that the distances were 29.9 mm at the tragus, 31.0 mm at the midpoint, and 31.3 mm near the ear. It was similar to our study that the mean distance between the ala-tragus line and the occlusal plane was higher at the upper tragus compared to the middle, and the middle showed a greater distance than the lower ala of the nose.

Considering orthodontic treatment and non-orthodontic treatment, sagittal jaw relationship is an important relationship for orthodontic treatment planning. The measurements of orthodontic planning in conventional methods have been used the bony landmarks in lateral cephalometric radiographs such as the inclination between the AB plane and the occlusal plane or the inclination between SN plane and the occlusal plane [24]. The angle between AB plane and the occlusal plane has a strong correlation with vertical and horizontal growth directions [37]. Currently, bony landmarks in 3D method provide more accuracy than 2D method. The distance between the perpendicular projection of points A and B measured on the Frankfurt horizontal plane showed higher reliability in skeletal class determination on CBCT than 2D method [38]. However, there is a limitation of research on the parallelism between the ala-tragus line and the occlusal plane in orthodontic patients because the bony landmarks have been widely used in orthodontic treatment planning. An ala-tragus line is the soft tissue landmark and has been widely used in prosthetic planning. The results of this study have shown that the mean angles in both methods were not found to be significantly different between non-orthodontic and orthodontic treatment volunteers (*p* > 0.05). The mean distances in both methods were not found to be significantly different between non-orthodontic and orthodontic treatment volunteers (*p* > 0.05). From our study, the orthodontic treatment was not affected to measurement the angle and distances between the ala-tragus line and the occlusal plane.

Although the results of this study agreed with the previous findings [33–38] that the ala-tragus line was not parallel to the occlusal plane. In clinically, the ala-tragus line was still deemed a reliable landmark for occlusal plane determination such as full denture fabrication. This reliability was attributed to the differences in angles and distances between the ala-tragus line and occlusal plane observed in both methods. In addition, the percentage difference of angles in conventional method were reported to be significantly higher by 13.61–21.58% (*p* < 0.05) compared to the 3D method. The percentage difference of distances in the conventional method were reported to be significantly greater than the 3D method by 4.73–7.51%. (*p* < 0.05) in both non-orthodontic and orthodontic treatment volunteers.

Clinically, to implement the results of this study, there are some concerning about the limitation of the study such as the race of the volunteer, the anatomy of the Asia people and other may be different and the number of the volunteers. Further studies are required to confirm these results.

Consequently, the study results support the use of the ala-tragus plane for reestablishing the occlusal plane in patients. Future investigations should focus on exploring more robust references for achieving optimal prosthetic and orthodontic outcomes.

## Conclusions

Within the limitations of this study, it can be concluded that both conventional and digital methods for establishing the occlusal plane are not parallel to the occlusal plane. The occlusal plane and ala-tragus line in the conventional method and the 3D method were significantly different in terms of angles and distances in both non-orthodontic and orthodontic treatment volunteers. However, the deviation angle of both methods is approximately 13–20 degrees, which is clinically acceptable for occlusal plane establishment. The accuracy of both methods is still within the using in clinical implementation.

## Data Availability

Data is provided within the manuscript or supplementary information files.
